# Mechanical Property Evaluation and Prediction of Cementing Composites Blended with MK and UFA under High-Temperature Steam Curing

**DOI:** 10.3390/ma15196956

**Published:** 2022-10-07

**Authors:** Chao Liang, Yongming Xing, Xiaohu Hou

**Affiliations:** 1 College of Science, Inner Mongolia University of Technology, Hohhot 010051, China; 2 College of Civil Engineering, Inner Mongolia Technical College of Construction, Hohhot 010070, China; 3 College of Materials Science and Engineering, Inner Mongolia University of Technology, Hohhot 010051, China

**Keywords:** composite cement-based material, high-temperature steam curing, low-field nuclear magnetic resonance, nanoindentation, multi-fractal dimension, grey relevance prediction, compressive strength

## Abstract

In this paper, the influence of the substitution rate of metakaolin (MK) and ultrafine fly ash (UFA) on the hydration degree, the micromechanical properties, the pore size distribution, and the corresponding fractal dimension of composite cement-based material was investigated under high-temperature steam curing. Furthermore, Thermogravimetric, Nanoindentation, and low-field nuclear magnetic resonance tests were used to explore the influencing factors of pore size distribution and its corresponding multi-fractal dimension. Finally, the correlations among the pore size distribution, related fractal dimensions, and compression strength were analyzed. Results indicate that the MK-UFA cement ternary cementation system (TCS) can improve the compressive strength and fluidity of samples and enhance the hydration degree and micromechanical properties of the cementitious system. TCS effectively refines the pore size and increases microporosity. In addition, micropore and its fractal dimension have a stronger correlation with the compressive strength of composite cement-based materials. Furthermore, the micro-fractal dimensions can better reflect the essential characteristics of the composite cementitious system. The higher the degree of hydration of the cementitious system and the nanomechanical properties of the C-(A)-S-H gel, the lower the micro-fractal dimension. Finally, the GM (1,3) prediction model of compressive strength, micro-fractal dimension, and microporosity are established based on the grey relational theory.

## 1. Introduction

Due to the rapid growth of the cement industry, man-made and natural pozzolanic materials are increasingly used in precast and mass concrete projects such as prefabricated constructions and integrated urban plumbing corridors. In these projects, high-temperature steam curing is extensively applicated, which is an effective method affecting the hydration rate of cementitious materials and has a significant promoting effect on the microstructure and macroscopic mechanical properties of cementitious materials. However, it frequently results in a decrease in the ultimate strength and durable performance of the cementitious materials, such as salt resistance [1,2].

Pozzolanic materials can enhance the strength of cement-based materials while lowering the heat of hydration and cement production costs under high-temperature steam curing conditions, enabling the economic and ecological progress of concrete engineering [3,4,5,6].

One of the most commonly used pozzolans in the cement and concrete industry is metakaolin (MK), which is among the most abundant minerals. It can be acquired from the thermal decomposition of kaolinites. The behavior of clay minerals during calcination determines optimal activation of pozzolanic reactivity [7]. During the calcination of kaolin, its main mineral phase–kaolinite–is dehydroxylated into metakaolinite. Dehydroxylation of kaolinite commonly occurs between 400 and 800 °C, but the dehydroxylation temperature depends on many factors, such as heating conditions and the structural layer stacking order [8]. The disorder of kaolinites causes stacking faults, often described as low crystallinity. According to the results of Izadifar et al. [9], disordered kaolinites dehydroxylate at lower temperatures than well-ordered kaolinites. These results strongly indicate the necessity for characterizing the structure of dioctahedral 1:1 layer silicates in kaolins and clays as a critical parameter to predict optimized calcination conditions and resulting reactivity.

MK is an anhydrous aluminosilicate, which is a representative supplementary cementitious material with high pozzolanic reactivity and filling effect. The silicates provided by MK react with the portlandite (CH) of cement to produce a secondary C-(A)-S-H gel in the pozzolanic reaction [10,11]. Additionally, the aluminum in the MK structure reacts with carbonate to form additional aluminate ferrite monosulfate phases, which provide more nucleation sites for the hydration of the Portland cement [12,13]. By using nanoindentation and thermogravimetric analysis (TG/DTG), Wang et al. [14] and Hou et al. [15] proved that the pozzolanic material can effectively increase the degree of hydration and enhance the micromechanical properties of C-(A)-S-H gel, respectively, and found that nanoindentation was an effective method to determine the micromechanical properties of cement-based materials. Regardless of the various benefits of MK on the mechanical properties of cement-based materials, an obvious problem induced by MK addition is the degradation of workability due to its layered structure and high specific surface area [16,17]. One approach to overcoming the adverse effect is using ultrafine fly ash (UFA), with spherical-shaped particles, which modifies the workability of MK cementing material [18]. UFA is an alumina-rich pozzolan material with spherical-shaped particles and has high pozzolanic activity. However, the study of the mechanical properties and working properties of MK-UFA cement ternary cementing material is not systematic.

The macroscopic properties of composite cement-based materials are closely related to their pore structures [19,20]. Therefore, it is crucial to investigate the relationship between pore structure and mechanical properties. Sabir et al. [21] have studied MK as a pozzolan material for concrete. According to the results, the total pore volume having negatively correlated with the compressive strength was found to decrease with an increase in curing time and increase with increasing MK content. Shanahan et al. [22] examined the influence of MK and slag on the mechanical properties of concrete. The findings revealed that MK and slag particles presented high pozzolanic activity and could decrease the porosity and refine pore diameter, resulting in compact microstructures and enhancement of macroscopic mechanical properties. This suggests that the morphology and connectivity of the pore structure are complex and disordered, and the type of pozzolanic material has different effects on the pore structure. Therefore, it is necessary to investigate the macroscopic mechanical properties of cement-based materials by pore size distribution.

The fractal theory is another effective method that has been proved to characterize the complex pore structure, and the concept of fractals is relevant to the study of the structural characterization of porous media involved in mechanical properties. Benoit, B et al. [23] first introduced fractal geometry as an important method to explain the irregular structures of materials by approximating them to regular patterns. Ji et al. [24] constructed the space-filling process model using the fractal dimension of pore size and found that the higher the fractal dimension, the looser the microstructure of the cement-based material. Charlotte et al. [25] employed the NMR technique to investigate the pore structure of the cement-based material. The results revealed that NMR is a non-destructive method to test the pore structure, which, when combined with the fractal theory, could suitably describe the various features of the pore structure and establish the relationship between the fractal dimension and other experimental parameters. Jianhua et al. [26] researched the pore network in stone powder cement tailings. The study confirmed that the fractal dimension was positively correlated with the stone powder content and compressive strength. At present, the pore structure and their corresponding fractal dimensions were addressed as a whole in these studies. There has been limited research on the division of pores into different pore sizes to investigate how multi-fractal dimension influence mechanical properties. Furthermore, there have been few studies on the microstructure of MK-UFA ternary cementitious systems (TCS) by multi-fractal dimension.

The main objective of this study is to investigate the compressive strength, fluidity, hydration degree, micromechanical properties, and pore size distribution characteristics of the TCS under high-temperature steam curing conditions. Furthermore, the factors influencing the pore size distribution and related multi-fractal dimensions are analyzed by hydration degree and C-(A)-S-H gel phase nanomechanical properties. Finally, the correlation between the compressive strength, micro-fractal dimensions, and microporosity was established based on the grey correlation prediction model. A better understanding of the mechanism of strength enhancement and improving workability was achieved by optimizing the content of MK and UFA.

## 2. Materials and Methods

### 2.1. Raw Materials

Ordinary Portland cement with 42.5 grade (Jidong Co. Ltd., Inner Mongolia, China) complies with Chinese national standard GB175–2007. In this study, ISO-standard sand was used. Metakaolin (Sanxing Co. Ltd., Inner Mongolia, China) has an average particle size of 1.52 µm and a surface area of 10,000 m^2^/kg based on the manufacturer’s data. Ultrafine fly ash (Toketuo Power Generation Co., Ltd., Inner Mongolia, China) was used. The surface area of fly ash is an average particle size of 4.51 µm and 700 m^2^/kg based on the manufacturer’s data. Table 1 summarizes the chemical compositions of raw materials which were measured by X-ray fluorescence spectrometry (XRF). The superplasticizer in this study was the polycarboxylate superplasticizer. The test dosage of the superplasticizer was uniformly 0.1% of the mass of the cementitious material.

### 2.2. Preparation of the Samples

Four MK replacement contents of 0%, 10%, 20%, and 30% were prepared in metakaolin-cement binary cementing systems (BCS) and designated as CM00, CM10, CM20, and CM30, respectively. In TCS, MK and UFA replaced cement in a ratio of 2:1 and were termed CMF105, CMF2010, and CMF3015. An extra number on the furthest right side of the letters is required to denote the curing time. For instance, CMF20103 implies that the sample contained 20% MK and 10% UFA and was cured for 3 days in high-temperature steam curing conditions. The water–binder ratio in each sample was 0.18, while the sand–binder ratio was 3, as shown in Table 2.

During sample preparation, binder and sand were mixed at a low speed for 2 min. Then, water was injected at a high pace for 5 min. The fluidity of the samples was measured complying with methods for testing uniformity of concrete admixture (GB/T 8077–2019). Following the fluidity test, the mixed slurry was cast into dimensions of 40 × 40 × 40 mm for TG–DTG, Nanoindentation, NMR, and compressive strength testing. After 24 h, the samples were de-molded and placed in a high-temperature steam box for 1, 3, and 6 days. The temperature was 70 °C, with a relative humidity of more than 90%. When the curing period was completed, the samples were placed in a vacuum saturation apparatus for 4 h before being removed for NMR testing. Finally, mechanical tests were performed on the samples, and a part of the sample debris was submerged in isopropanol for 1 day before being vacuum dried for the TG–DTG analysis.

For the nanoindentation test, the samples were cut into discs with a diameter of 2 cm and a thickness of 1 cm, embedded in epoxy, and polished to obtain flat surfaces. To obtain flat surfaces in all the samples, five types of silicon carbide abrasive papers, namely #400, #800, #1000, #1600, and #2400, were chosen for coarse grinding. To remove surface impurities, the samples were ultrasonically cleaned for 10 min. Next, a grinding and polishing machine (Buehler EcoMet 250) was employed for fine polishing. For successive fine polishing for 15 min, three types of polishing liquids (with particle sizes of 6, 3, and 1 µm) with matching polishing cloths were used. After grinding and polishing, all the samples were cleaned in the ultrasonic cleaner for 5 min and finally stored in a drying oven.

### 2.3. Test Methods

The fluidity of the samples was measured as described in methods for testing uniformity of concrete admixture (GB/T 8077–2019). The compressive strengths were evaluated under high-temperature steam curing conditions where three specimens were tested for mixture and age, and the loading rate was kept at 10 KN/S. For the TG/DTG tests, the crumbs were pulverized to pass through a 45 µm filter and were performed using the Mettler–Toledo simultaneous thermal analysis apparatus (TGA/SDTA 851) (Mettler-Toledo instrumenstruments Co., Ltd., Shanghai, China). An alumina crucible was used to heat the samples from 5 to 1000 °C at a rate of 10 °C/min in an N_2_ environment.

Nanoindentation tests were performed using the Ti950 Triboindenter nanoindenter (Bruker Co., Ltd., Karlsruhe, Germany). The load control mode was adopted in which the loading-unloading cycle test was carried out for each measuring point in turn. The specific process is as follows: at a constant rate of 0.2 µN/s, the load is increased until the maximum value of 2 µN is attained. Then, this load was maintained for 5 s before unloading at the same constant rate. In the nanoindentation test, due to the heterogeneity of the composite cementitious system, a 7 × 7 indentation matrix was set with a spacing of 8 µm in each sample matrix. According to the Oliver–Pharr principle [27,28], the indentation modulus (E) at each measuring point is calculated as Equation (1):

(1) $$\frac{1}{E_{r}}=\frac{1-v^{2}}{E}+\frac{1-v^{2}}{E_{i}}$$

where: E: Elastic modulus of cement-based materials; v: Poisson ratio; $E_{i}$: indenter elastic modulus, GPa; For diamond indenters, $E_{i}=1141\mathrm{GPa}$,$v_{i}=0.07$; for cementitious materials v=0.25; Studies have shown that $E_{r}$ of cementitious materials meet Gaussian distribution [28].

In this paper, NMR testing was performed using the MiniMR-60 magnetic resonance imaging analysis equipment (Niummag Electronic Technology Co., Ltd.,Suzhou, China). The primary magnetic field of the device was 0.51 Tesla, and the H-proton resonance frequency was 21.7 MHz. $T_{2}$ is relaxation time and directly proportional to the pore size, as expressed in Equation (2):

(2) $$\frac{1}{T_{2}}=\rho_{2}\frac{S}{V}$$

where $\rho_{2}$ is the surface relativity strength, μm/ms; S/V is the surface area to volume ratio of the pores, $\mu m^{-1}$.

The pore fractal dimensions can be derived from the NMR data using the fractal geometric principle. According to previous studies [26,29,30], the fractal geometry expression of pore size distribution was expressed as Equation (3):

(3) $$S_{v}=\frac{r^{3-D}}{r_{\max}^{3-D}}$$

By taking the logarithm of both sides of Equation (3), the fractal geometric expression of the NMR $T_{2}$ spectrum of the sample can be obtained as Equation (4):

(4) $$\mathrm{lg}(S_{v})=(3-D)\mathrm{lg}(T_{2})+(D-3)\mathrm{lg}(T_{2\max})$$

## 3. Results and Discussion

### 3.1. Working and Macro-Mechanical Performance Analysis

**Figure 1:**
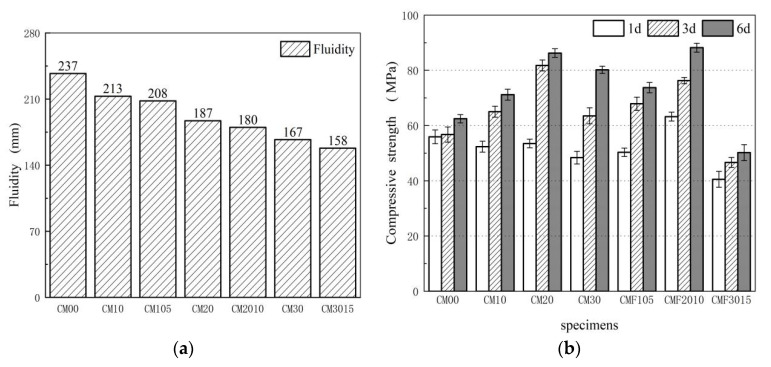
(**a**) Fluidity performance and (**b**) compressive strength of blend composites.

Figure 1a shows the fluidity of the composite slump as a function of MK and UFA content. As the rate of MK substitution increased, the fluidity of each sample decreased. The fluid ability of CM00 was 1.42 times that of sample CM30. The fluidity of TCS is improved to a certain extent as compared to the BCS. The fluidity of CMF2010 is 7.78% higher than that of CM30. The reason is that even though, similar to MK, UFA has a large specific surface area, UFA, MK and cement have different particle size distributions, which can optimize particle size distribution, increase free water content, and ultimately improve the fluidity of mixed slurry to a certain extent. Furthermore, the spherical particles of UFA improve fluidity. The fact that the fluidity of CMF105 is close to that of CM10 proves this conclusion.

Figure 1b displays the compressive strength trend of composite cement-based materials. As can be observed, all the mixtures blended with MK show a decreased compressive strength on day 1. Moreover, the higher the content, the lower the early strength. This is indicative of the low pozzolanic reaction of MK in the early stages of hydration. MK could significantly promote compressive strength on the third day. The compressive strength of CM103, CM203, and CM303 increased by 10.35%, 40.1%, and 31.11%, respectively, as compared to CM003, with CM203 attaining the maximum compressive strength of 81.78 MPa. It was attributed to the high activity of MK and ease of excitation at high-temperature steam conditions, respectively. The compressive strength of each sample with MK increased slowly after 3 days, with CM203 showing an increase of 4.65%. The increase in compressive strength of the blended paste with MK mainly occurred in the first 3 days, with an optimal replacement rate of metakaolin of 20%, and a curing time of 3 days.

Another finding was that in the TCS, the compressive strengths of CMF20103 were 76.31 MPa at day 3, which were 6.81% lower than CM203. However, at a later age (on the sixth day), the compressive strengths were 1.04 and 1.1 times higher than CM206 and CM306. This indicates that the TCS can further improve the compressive strength and increase the substitution rate of pozzolanic materials. However, its strength increased slowly compared with BCS, which can explain why UFA exhibits a relatively lower pozzolanic reaction. Since the compressive strength of CMF3015 is significantly lower than other samples due to the dilution effect [31], it was not analyzed in this paper.

As compared to the BCS, the TCS with a replacement level of up to 30% can improve the fluidity and ultimate compressive strength. It is assumed that the strength of cementitious materials is linked to the hydration degree, pore structure, and nanomechanical properties of the complex cementitious materials, which will be discussed in the following sections.

### 3.2. Hydration Analysis

**Figure 2:**
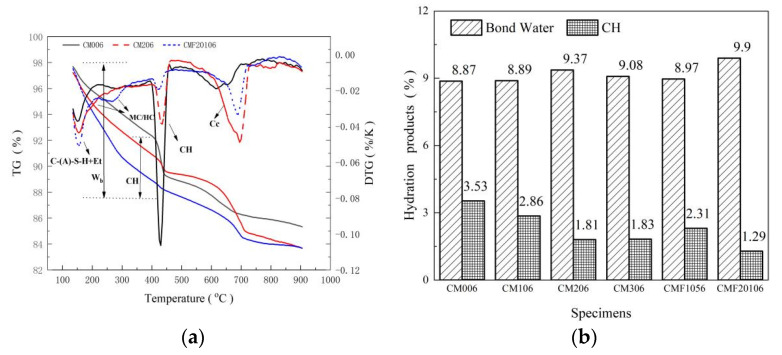
(**a**) Thermogravimetric analyses curve (TG and DTG) and (**b**) hydration products analysis.

Hydration is associated with the pore structure of cement-based materials, which has a considerable impact on the macroscopic mechanical properties. Aluminum-rich pozzolan materials, such as MK and UFA, react with the CH crystal formed during cement clinker hydration to form a novel C-(A)-S-H gel. Furthermore, the large proportions of amorphous silico-aluminates in MK and UFA will consume part of the CaCO_3_ in the pozzolanic reaction, increasing the alumino-silicate hydrates carboaluminate (Mc, and Hc). With the continuous dissolving of calcite, Hc is converted to Mc. That new hydration production is considered to fill the voids and benefit the refinement of pores. As shown in Figure 2a, sample CM006 shows a small signal for Hc and Mc, which becomes more evident when MK and UFA are incorporated. Thus, a pozzolanic reaction can increase the type and quantity of hydration products. Both pozzolanic and cement hydration reactions form bound water, which may be used to determine the hydration degree of composite cementitious materials.

As illustrated in Figure 2b, MK can effectively promote bound water content. The bound water content of CM106, CM206, and CM306 increased by 0.23%, 5.64%, and 2.37%, respectively, as compared to CM006. Sample CM206 has the highest bound water content. It is observed that incorporating MK promotes cement hydration and has higher pozzolanic activity, which can offset the dilution effect. However, with the further addition of MK, the bound water content is reduced. This is due to the use of a large amount of MK instead of cement, which reduces the content of cement clinker and reduces the pH value of the mixed slurry, which hinders the pozzolanic reaction of MK [11]. The CMF20106 has a 30% cement substitution rate. However, its combined water content is 5.65% and 9.03% higher than that of CM206 and CM306. Therefore, the TCS can better promote the pozzolanic reaction and the nucleation effect because UFA has a high content of calcium oxide (as shown in Table 1) and high specific surface area. It not only has a positive effect on forming hydration products, such as C-(A)-S-H, Hc, and Mc, but also increases the pH value of the slurry solution by depolymerization reaction, promoting the pozzolanic reaction between MK and UFA, thus, improving the gel content. The same conclusion was obtained for sample CMF1056.

CH crystals are coarse and have a certain orientation in the matrix. Although their elastic modulus is large, it readily causes cracks, and their uneven distribution causes the micro-structure to become loose. The CH crystals content are calculated as a substantial mass decrease in the temperature range of 400–500 °C in the TG curve, in Figure 2a. As indicated in Figure 2b, the CH crystal of CM206 is only 48.73% of CM006. Due to the pozzolanic reaction and dilution effect, the incorporation of MK significantly reduces the CH crystal. Furthermore, the TCS is more effective in reducing the CH crystal compared with BCS. For CMF20106, the content of CH crystal is only 1.29 %. It indicates that in the TCS, a higher degree of pozzolanic reaction can be carried out.

### 3.3. Micro-Mechanical Properties Analysis

**Figure 3:**
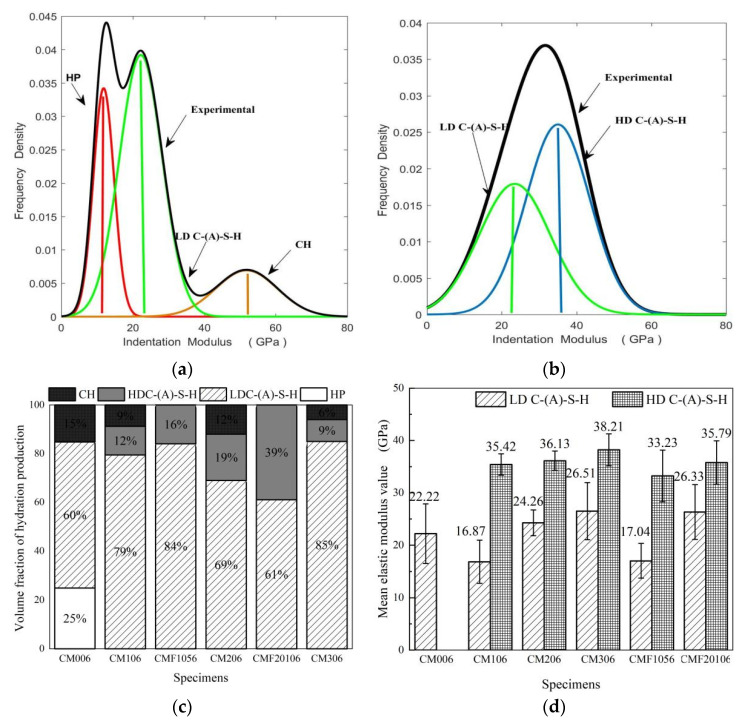
Nanoindentation point Gaussian fitting results of sample (**a**) CM006 and (**b**) CMF20106, and (**c**) Volume fraction and (**d**) Gaussian fitting average elastic modulus value of C-(A)-S-H.

Using statistical nanoindentation, researchers have found the range of elastic modulus values of phases in cement-based materials, including HP (0–12 GPa), LD C-(A)-S-H (13–25 GPa), HD C-(A)-S-H (26–38 GPa), and CH (>38 GPa) [28,32]. The microscopic mechanical properties of C-(A)-S-H gels, especially the HD C-(A)-S-H gel, effectively enhance the macroscopic performances of cementitious composites [32]. The Gaussian multi-peak fitting results of samples CM006 and CMF20106 are shown in Figure 3a,b.

Figure 3c,d reveal the variation of the percentage stacking chart of hydration products and the average elastic modulus of C-(A)-S-H gel with the MK and UFA replacement ratio. As shown in Figure 3c, the content of the defect phase (HP) and CH phase decreases gradually with the increase in the MK replacement rate, with only CM006 fitting out the defect phases. This indicates that MK effectively increases the gel phase, consumes CH crystals, and repairs the matrix cracks, which is consistent with the TG/DTG result in 3.2. This effect is more pronounced in the TCS. For CMF20106, in Figure 3b, only C-(A)-S-H phases are fitted, and the HD C-(A)-S-H phase is as high as 39%, which is significantly higher than CM20. In Figure 3d, MK and UFA can also increase the elasticity of the C-(A)-S-H phase. Sample CM006 does not fit out the HD C-(A)-S-H phase in Figure 3a, and the average elasticity of HD C-(A)-S-H of CMF20106 is higher than CM106, CM206, and CM306 14.49%, 5.73%, and 2.34%, respectively. Therefore, the results reveal that the TCS can improve the uniformity of the matrix and increase the nanomechanical properties of the C-(A)-S-H gel.

### 3.4. NMR Pore Structure Characteristics and Multi-Fractal Dimension Analysis

#### 3.4.1. NMR Pore Structure Characteristics Analysis

**Figure 4:**
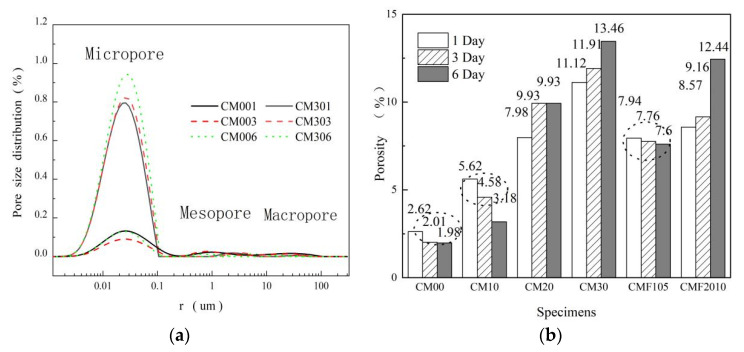
(**a**) NMR pore size distribution and (**b**) porosity of blended paste composites.

The pore structure is a crucial criterion for evaluating the mechanical properties of composite cement-based materials. In this paper, the impact of curing age and the replacement rate of MK and UFA on the pore size distribution characteristics of the composite cementing system was analyzed based on the NMR test results. According to Jianhua et al. [26], the interlayer water, adsorbed water, capillary water, and free water divided by pore size are represented by micropores, mesopores, and macropores, respectively. Their pore size ranges and relaxation times are as follows: micropores (pore radius: 0–100 nm, relaxation time: T_2_ < 2.5 ms), mesopores (pore radius: 100–10,000 nm, relaxation time: 2.5 < T_2_ < 50 ms), macropores (pore radius: >10,000 nm, relaxation time: T_2_ > 100 ms) [25,26,33]. Based on this conclusion, as shown in Figure 4a, the pores are distributed and named.

The porosity of the samples showed a distinct trend with age due to the substitution rates of MK and UFA in Figure 4b. As confirmed by previous studies [25,34], the porosity decreases with curing age for samples with low substitution rates, such as CM00, CM10, and CMF105. Physically, the hydration process is regarded as the filling process of the hydration products into the space originally occupied by water and unhydrated particles. The porosity exhibited an entirely different trend with curing time for samples with higher substitution rates of pozzolanic materials such as CM20, CM30, and CMF2010. As shown in Table 3, although the porosity of CM306 is higher than that of CM301, its mesoporosity and macroporosity are 30.52% and 67.74% lower than those of CM301, respectively. The increase in porosity of higher substitution rates samples is primarily due to the content of micropores, and the downward trend for mesopores and macropores is the same as those of samples with lower substitution rates. Table 3 further shows that the mesopores and macropores of CM306 are 16.41% and 68.75% lower than that of CM006, respectively. Therefore, the addition of MK is beneficial to the pore size refinement of samples. Furthermore, the pore size distribution characteristics accurately reflect the change law of the pore structures of cement-based materials.

**Figure 5:**
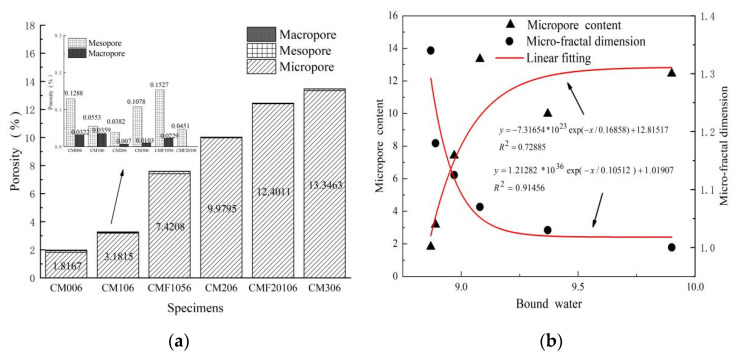
(**a**) Pore size distribution of paste blends and (**b**) relationship between micropority and bound water.

In this paper, 6-day samples were used as the research object to further explore the impact of MK and UFA content on the pore size distribution characteristics. As shown in Figure 5a, with the increased substitution rates of the MK and UFA, the microporosity increased, while the mesoporosity and macroporosity gradually decreased. There was no macropore for CMF20106. It is observed that with the increase in the substitution rates of pozzolanic materials, the pore size refinement is better and more obvious. Feng et al. [35] found that the micropore content reflected the hydration degree of cement-based materials. Therefore, a nonlinear fit was made between microporosity and the bound water content of the 6-day samples. As shown in Figure 5b, the correlation was only 0.72885. Moreover, the hydration degree of composite cement-based materials reflects microporosity only to a certain extent and several factors affect the microporosity.

As indicated in Figure 5a, samples CMF20106 and CM306 had the same substitution rate of pozzolanic material. The content of micropores and mesopores was 7.08% and 58.16% lower than CM306, respectively. Therefore, the pore size distribution is still affected by the type of the pozzolan material. The layered structure of MK increases microporosity, while for the TCS, the particle gradation is optimized, improving the compactness of the matrix. Thus, the content of micropores can be reduced to a certain extent. Furthermore, in the high-temperature steam curing environment, the free water in the matrix expands and then overflows, leading to an increase in microporosity [36]. Thus, microporosity is affected by the degree of hydration, the number and type of pozzolanic materials, and the curing method.

The hydration products, unhydrated particles, and bulk CH crystals influence the content of mesopores. Therefore, the mesopores decrease with the increase in the mineral admixture material due to the pozzolanic and filling effects. Macropore did not demonstrate apparent regularity, consistent with Jin et al. [34]. The reason is as follows: mesopores and macropores will be affected by many external factors, such as bubbles in the stirring and cracks in the matrix as the pore size increases. In addition, the content of macropores and mesopores is low, less than 0.2% of the total porosity, as shown in Figure 5a. Therefore, they are random. In summary, mesopores and macropores could not reflect composite cement-based materials’ essential characteristics.

#### 3.4.2. NMR Multi-Fractal Dimension Analysis

**Figure 6:**
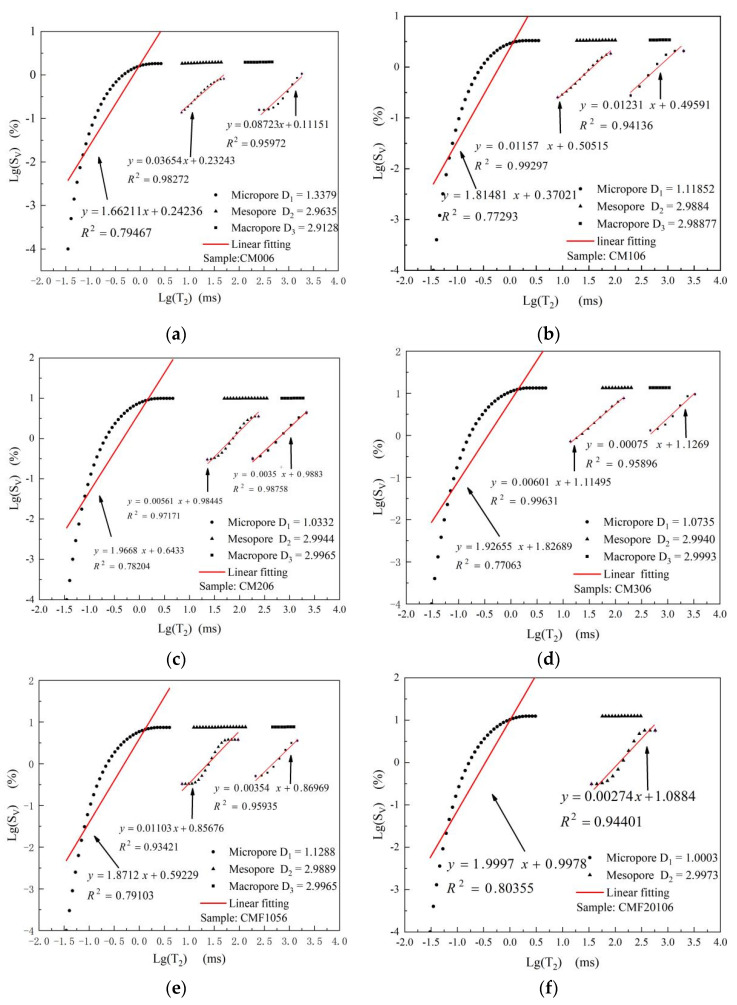
(**a**–**f**) Multi-fractal characteristics for blended composites.

Fractal dimension is an effective quantitative method for evaluating the complexity and heterogeneity of pore structures based on the fractal theory [37]. According to Qiang et al. [33], determining the complexity of the pore structure by using only the total poro–fractal dimension is challenging. Therefore, in this paper, as shown in Figure 6a–f, the multi-fractal dimension of the 6-day samples was analyzed based on NMR data and the aforementioned division of pore sizes. The micro-fractal dimension (pore radius: 0–100 nm) of each sample exhibited a high correlation, reaching more than 0.77063, indicating that the correlation was strong, and the fitted lines were reliable. Based on the above linear regression model, the f-test method was used to test the significance of the linear regression relationship [38], and the significance level was set as 0.05. for the mesopores (pore radius: 100–10,000 nm), and macropore (pore radius: >10,000 nm), which will be affected by many external factors. Its distribution is random and not interesting to study. Therefore, this paper only analyzes the micro-fractal dimension.

To analyze the relationship between the substitution rate of pozzolanic materials and the multi-fractal dimension and to explore the factors affecting the fractal dimension, the multi-fractal dimension method was utilized. The nonlinear fitting results between the bound water content and micro-fractal dimension are shown in Figure 5b. The correlation states 0.91456 is higher than that of the micropore content. This indicates that the micro-fractal dimension significantly correlates with the matrix’s C-(A)-S-H gel content. The reason is that with the progress of the pozzolanic reaction, there is a large number of new C-(A)-S-H gel, which increases the type and quantity of hydration products, and then simplifies the inter-lay of C-(A)-S-H gel and tightens the packing of the binder particles to form a dense internal microstructure. However, the micro-fractal dimension did not increase with the continuous improvement of the MK replacement rate from 20% to 30%. This phenomenon was observed in the sample CMF20106 too, which has the same replacement rate as CM306, but different types of pozzolanic materials. This further illustrates that the number and type of pozzolanic materials have a weaker effect on the micro-fractal dimension. This is consistent with the Jianhua et al. [26] conclusion on tailings content and micro-fractal dimension.

The nanomechanical properties of the C-(A)-S-H gel phase significantly affect the micro-fractal dimension. The micro-fractal dimension is negatively correlated with the nanomechanical properties of the C-(A)-S-H gel phase. The micro-fractal dimension of CMF20106 is 1.0003, which is lower than CM306 and CM206. In the meantime, CMF20106 also has the highest content of HD C-(A)-S-H gel. This is because the pozzolanic reaction of MK increases the number of gels per unit volume and then increases its bulk density. In addition, the pozzolanic ash effect of MK decomposes the bulk CH crystals into CH nanoparticles to fill the gel pores, which is also helpful in improving the nanomechanical properties of C-(A)-S-H gel, especially in generating the HD C-(A)-S-H gel phase [39].

The conclusion is that the increase in hydration products and their packing density decrease the complexity inter-lay of C-(A)-S-H gel and then reduces the value of the micro-fractal dimension. Therefore, the micro-fractal dimension can more accurately reflect the essential characteristics of the microstructure of composite cementitious materials.

### 3.5. Correlation Evaluation and Prediction of Macroscopic Mechanical Properties with Pore Distribution and Fractal Dimension

#### 3.5.1. Correlation Analysis of Macroscopic Mechanical Properties with Pore Size Distribution and Multi-Fractal Dimension

**Figure 7:**
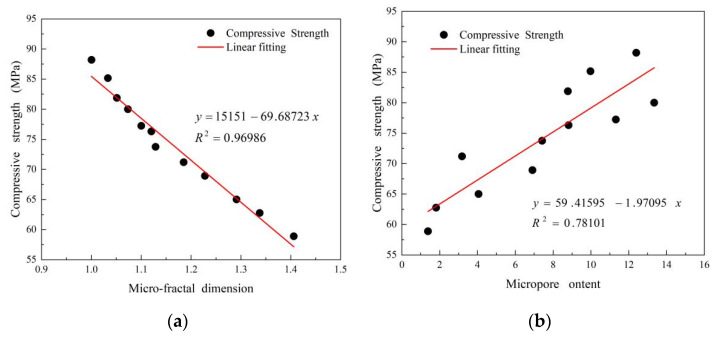
Relationship between (**a**) compressive strength and microporosity and (**b**) compressive strength and micro-fractal dimension.

In Figure 7, the scatter diagrams of microporosity and their corresponding fractal dimensions and compressive strength were drawn, and regression analysis was carried out. The micro-fractal dimension was negatively correlated with the compressive strength and had the correlation coefficient of 0.96986, which was higher than that of microporosity. The micro-fractal dimension of CM20106 was the lowest value of 1.0003, which corresponds to the highest compressive strength of 88.26 MPa. It has been proven that micro-fractal dimension can more accurately reflect the compressive strength of composite cement-based materials. This also proves the correctness of the conclusion of Section 3.4.2. Furthermore, this illustrates that the TCS is beneficial to the improvement of macroscopic mechanical properties.

#### 3.5.2. Grey Relational Prediction Model GM (1,3)

Jingqiu et al. [40] proposed the grey correlation prediction model based on the grey correlation analysis method. The model has been widely applied in the fields of economy and construction engineering in recent years. Vps et al. [41] provided a comprehensive summary of the grey correlation analysis theory and described how differential equations obtained by the grey correlation analysis method can represent certain characteristics of research problems. The following is a summary of the concept of establishing a grey relational prediction model:

(1) The grey correlation analysis is used to obtain a series of regular numbers based on a small amount of irregular original experimental data.
(2) The parameter values in the differential equation are determined according to the differential Equation (5) and the least square method.

(5) $$\frac{dx}{dt}+ax=b_{1}x_{2}+b_{2}x_{3}$$

(3) The mathematical expression of the differential equation, which is the prediction model, is obtained.

Based on the grey correlation analysis method and the data from the 3-day and 6-day samples, GM (1,3) was utilized to establish the grey correlation prediction model of compressive strength, micro-fractal dimension, and microporosity of composite cement-based materials under high-temperature steam curing. The parameter values of the differential equation in Equation (5) are obtained by MATLAB mathematical software and test data.

$$u=\left[\begin{array}{l} a\\ b_{1}\\ b_{2} \end{array}\right]=\left[\begin{array}{l} 2.71\\ 36.71\\ 0.81 \end{array}\right]$$

Therefore, the prediction model is Equation (6):

(6) $$x(t+1)=[88.26-36.71(3-x_{2})+0.81x_{3}]e^{-2.71}+[36.71(3-x_{2})-0.81x_{3}]$$

where: $x_{2}$: micro-fractal dimension; $x_{3}$: microporosity.

The above calculation, Equation (6), predicted compressive strength, and the results are shown in Table 4. The average relative error of the predicted value is 1.91%. The conclusion is that the compressive strength of cement-based materials corresponding to micro-fractal dimension, and microporosity can be accurately predicted according to the GM (1,3) model.

## 4. Conclusions

In this paper, the bound water content, the pore size distribution characteristics, and multi-fractal dimension of the MK-UFA cement ternary cementitious system were studied by TG/DTG, nanoindentation, NMR, and the fractal dimension theory. Additionally, micropores, mesopores, macropore and their corresponding correlation with the multi-fractal dimensions with macroscopic mechanical properties and microstructure were obtained and evaluated. Finally, the grey correlation prediction model was established, the specific conclusions of which are as follows:

1. Under high-temperature steam curing conditions, the compressive strength and fluidity of the MK-UFA cement ternary cementation system are improved when compared to the binary cementitious system (for example, the fluidity of CMF2010 is higher than that of CM30), effectively improving the hydration degree of the matrix and the elastic modulus of the C-(A)-S-H phase. Among them, the best replacement rate for metakaolin is 20%, and that for ultrafine fly ash is 10%. In the binary cementation system, the optimal replacement rate is only 20%.

2. The pore size distribution analysis based on NMR experiments revealed that substituting cement with MK and UFA can increase the porosity of composite cement-based materials but can effectively refine the pore size. Microporosity increases with the replacement rate of MK and UFA. Moreover, microporosity is affected by the degree of hydration, and the number and type of supplementary cementing materials (SCM). However, the mesoporosity and macroporosity decrease with the increase in the replacement rate of MK and UFA but do not show regular changes.

3. In the analysis of the multi-fractal dimension, an obvious correlation is observed between the micro-fractal dimension and the microstructure of the composite cementitious system. The micro-fractal dimension mainly reflects the bulk density and regularity of the microstructure. To be specific, the higher the hydration degree of the cementitious system and the nanomechanical properties of the C-(A)-S-H phase, the lower the micro-fractal dimension.

4. Micro-fractal dimensions can more preferably reflect the compressive strength of composite cement-based materials, and the relationship is inversely proportional. This further proves that the micro-fractal dimension can better characterize the essential characteristics of composite cement-based materials.

5. The GM (1,3) prediction model was established based on the grey correlation theory. The prediction model of the micro-fractal dimension and microporosity, and compressive strength was obtained, and the accuracy was 1.94%.

## Figures and Tables

**Table 1:** Chemical composition of raw materials wt.

Raw Material	CaO	SiO_2_	MgO	Al_2_O_3_	Fe_2_O_3_	Na_2_O	LOI
Cement	62.68	21.28	3.61	4.82	4.34	0.41	2.86
Metakaolin	0.2	50	0.09	46	2.6	0.4	0.71
Fly ash	17.6	65.67	0.08	6.84	4.59	1.7	3.88

**Table 2:** Mortar mix proportions wt.

Sample	PC	MK	UFA	Sand	Water
CM00	1.00	-	-	3.00	0.18
CM10	0.90	0.10	-	3.00	0.18
CM20	0.80	0.2	-	3.00	0.18
CM30	0.70	0.30	-	3.00	0.18
CMF105	0.85	0.10	0.05	3.00	0.18
CMF2010	0.70	0.20	0.10	3.00	0.18
CMF3015	0.65	0.30	0.15	3.00	0.18

**Table 3:** Variation of pore size distribution with curing time.

Sample	Pore Category	1 Day	3 Day	6 Day
CM00	Total porosity	2.620	1.898	1.978
	Microporosity	2.008	1.411	1.817
	Mesoporosity	0.415	0.345	0.128
	Macroporosity	0.189	0.143	0.032
CM30	Total porosity	11.181	11.756	13.465
	Microporosity	10.997	11.585	13.346
	Mesoporosity	0.154	0.143	0.107
	Macroporosity	0.031	0.029	0.010

**Table 4:** Prediction Value and Error check.

Code	Compressive Strength Value (MPa)		Residual (MPa)	Relative Error (%)
	Test Values	Predicative Values		
1	88.26	88.26	0.00	0.00
2	85.15	80.82	−4.33	4.33
3	81.81	79.29	−2.58	2.58
4	80.01	81.56	1.56	1.56
5	77.23	79.12	1.89	1.89
6	76.38	76.94	0.64	0.64
7	73.75	75.60	1.85	1.85
8	71.18	70.47	−0.71	0.71
9	68.92	71.82	2.90	2.90
10	65.43	67.41	2.41	2.41
11	62.75	64.20	1.45	1.45
12	58.89	61.53	2.63	2.63
Average Relative Error			1.91%	

## Data Availability

Not applicable.
